# Deep Eutectic Solvents in Polymeric Drug Carriers: Insights into Release Behavior and Functional Integration

**DOI:** 10.1002/open.202500332

**Published:** 2025-08-18

**Authors:** Nining Nining, Yoga Windhu Wardhana, Taofik Rusdiana, Hariyanti Hariyanti

**Affiliations:** ^1^ Doctoral Program in Pharmacy Universitas Padjadjaran Sumedang 45363 Indonesia; ^2^ Department of Pharmaceutical Technology Universitas Muhammadiyah Prof. DR. HAMKA East Jakarta 13460 Indonesia; ^3^ Department of Pharmaceutics and Pharmaceuticals Technology Universitas Padjadjaran Sumedang 45363 Indonesia; ^4^ Department of Pharmaceutical Chemistry Universitas Muhammadiyah Prof. DR. HAMKA East Jakarta 13460 Indonesia

**Keywords:** biocompatibility, deep eutectic solvents, functional excipients, polymeric drug delivery, release kinetics

## Abstract

Incorporating deep eutectic solvents (DES) into polymer‐based drug delivery systems (DDS) presents a novel approach to addressing persistent pharmaceutical issues, including low solubility, restricted bioavailability, and unregulated drug release. As environmentally friendly, adjustable, and biocompatible alternatives to traditional organic solvents and ionic liquids, DES possess distinctive physicochemical characteristics—such as strong hydrogen‐bonding ability, low volatility, and structural flexibility—that make them effective as functional excipients. This review consolidates a contemporary understanding of the formulation and performance of polymeric matrices containing DES, highlighting their capacity to alter drug release kinetics, improve solubility, and facilitate dual‐function systems like therapeutic DES (THEDES). A comprehensive overview of the various types of DES and polymeric carriers, their methods of integration, associated physicochemical effects, and drug release mechanisms is provided. Furthermore, the outline problems associated with stability, sterilization, and regulatory considerations. In addition, the most prospective future uses of DES–polymer systems is examined in stimuli‐responsive systems, 3D‐printed scaffolds, and advanced tailored medicine. This article supports the broader polite investigation of polymers and DES in polymeric systems in DDS as a significant step toward safer, more environmentally friendly, high‐efficiency pharmaceutical technologies.

## Graphical abstract

Incorporating deep eutectic solvents into polymer‐based drug delivery systems (DDS) offers a sustainable route to enhance solubility, modify drug release, and create dual‐function carriers. This review explores how DES influence polymer structures and DDS performance, highlighting future prospects in personalized, responsive pharmaceutical technologies. Created in BioRender. Nining, N. (2025) https://BioRender.com/kc12by9.

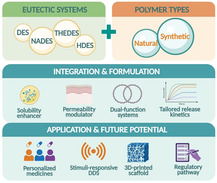

## 1 Introduction

In recent decades, DDS has advanced from conventional formulations to complex platforms which aim to solve problems such as low water solubility, the high molecular weight of therapeutic proteins and peptides, bioavailability, expedited systemic elimination, and unregulated release profiles of drugs.^[^ ^1^, ^2^ ^]^ Polymeric drug carriers have captured considerable attention from all the platforms due to their structural diversity, biocompatibility, and capability to modulate drug release kinetics via manipulation of matrix composition, design, and functional group incorporation.^[^ ^3^, ^4^ ^]^ Hydrogels, nanoparticles, films, and scaffolds serve as passive drug reservoirs and responsive matrices that modulate a given physiological milieu for controlled and targeted drug release.

Even with notable advancements, ensuring ease of formulation and safety while controlling complexity within drug release profiles remains an outstanding obstacle.^[^ ^5^ ^]^ Using cosolvents, plasticizers, and chemical enhancers to modify drug release within the body may pose questions regarding their toxicity, stability, and regulatory approval for incorporation into pharmaceuticals.^[^ ^6^ ^]^ Biocompatible and biologically safe approaches to increasing drug solubility and bioavailability are possible by developing ecologically “green” multifunctional solvent systems, which can also serve as environmentally friendly and adaptive solvents.

Abbott and coworkers first described DES in 2003, and they have since emerged as an exciting class of these solvents. The combination of a hydrogen bond donor (HBD) and a hydrogen bond acceptor (HBA) forms a mixture with considerably lower melting temperature than the constituent's components.^[^ ^7^, ^8^ ^]^ As with most eutectic solvents, deep eutectic solvents (DES) possess unparalleled low vapor pressure, a violation resistance to ignition, an outrageous ability to solubilize both hydrophilic and lipophilic compounds, and the low modification for a targeted chemical composition–all simultaneously.^[^ ^9^, ^10^ ^]^ As highlighted, the constituents of choline chloride, urea, lactic acid, and menthol, which are naturally sourced and biologically safe, allow for the synthesis of DES. These further provide remarkable compatibility with the principles of green chemistry.^[^ ^11^ ^]^

Recent studies indicate that integrating DES into polymer‐based DDS profoundly affects the composite's traits and the drug release kinetics. Based on a given formulation, DES can either promote the solubility of the active pharmaceutical ingredient (API), modulate the porosity of the polymer matrix through microstructural alterations, act as a plasticizer within the polymer network, or even serve as a bioactive coformulant.^[^ ^12^, ^13^, ^14^, ^15^ ^]^ An example would be specific, such as therapeutic DES (THEDES), which have shown capabilities to serve solvents and compounds with inherent biological activity, thus serving a dual purpose as excipients and APIs.^[^ ^16^, ^17^ ^]^

The specific objective of this review is to analyze and combine all known information relating to the use of DES in polymeric drug carriers, particularly their impact on drug release profiles and their bonds with the polymer matrices at structural and functional levels. The focus is on recent progress and representative case studies, which show the mechanisms and the actual suite of possibilities regarding DES‐based polymer systems in contemporary drug delivery. Moreover, it deals with some primary formulating problems and formulates some principles of their use to refine DES in developing pharmaceuticals for the next generation.

## 2 DES: Composition, Properties, and Biomedical Relevance

DES represent a class of tailor‐made solvents formed through the complexation of an HBA—typically a quaternary ammonium salt such as choline chloride—with an HBD, including compounds like urea, glycerol, lactic acid, or amino acids. The resulting eutectic mixture exhibits a melting point substantially lower than its components, primarily due to extensive hydrogen bonding and charge delocalization.^[^ ^8^, ^18^ ^]^ The initial systematic characterization of DES was completed by Abbott et al. in 2003, focusing on their low‐cost, tunable, and eco‐friendly features as alternatives to traditional ionic liquids.

**Figure 1:**
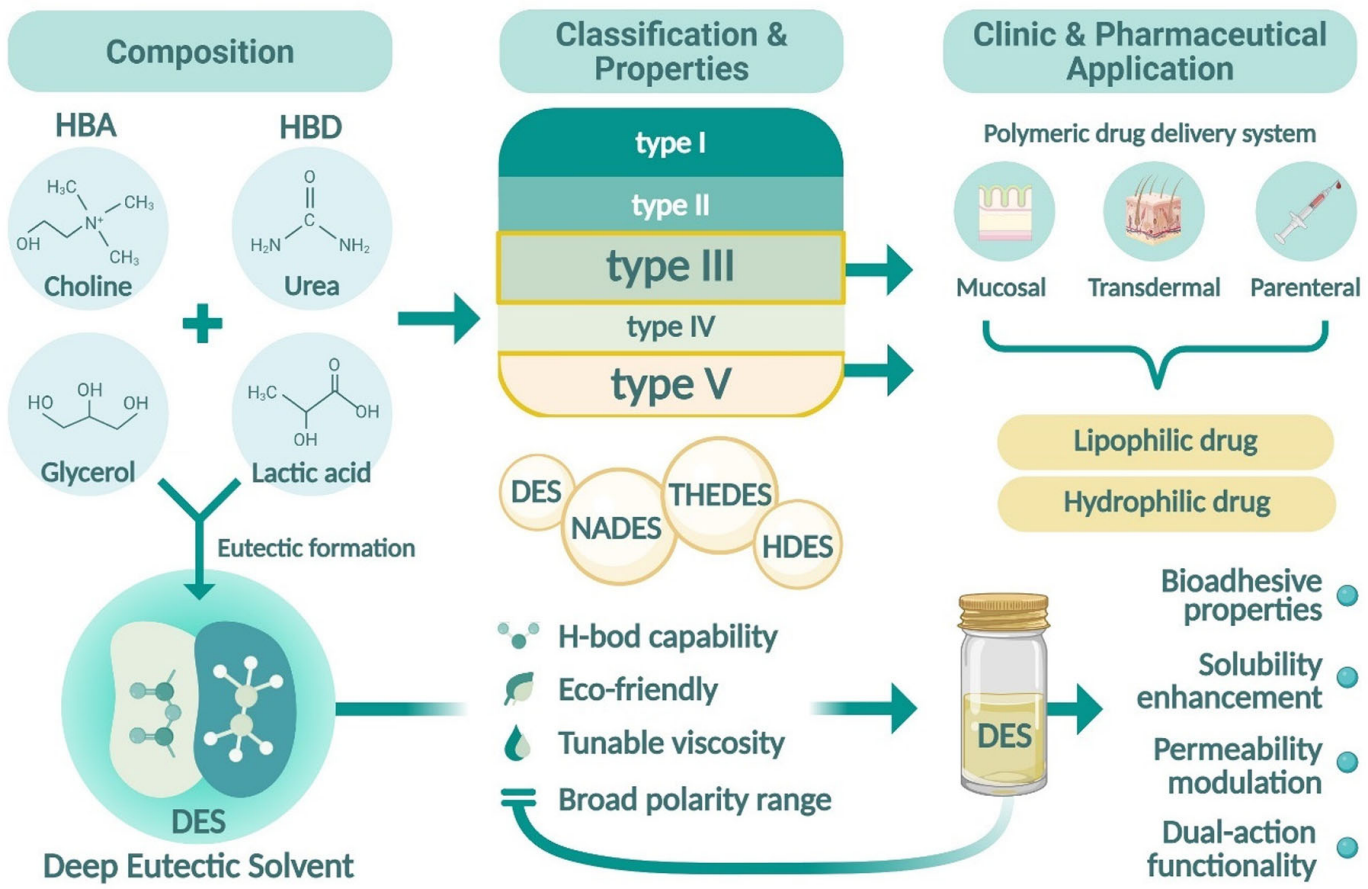
Overview of DES formation, their classification, key physicochemical properties, and biomedical applications such as enhanced solubility and permeability for drug delivery. Created in BioRender. Nining, N. (2025) https://BioRender.com/homg7yk.

From a constituent's perspective, DES can be classified into five types, I through V. Types I to III, usually consist of blends of quaternary ammonium salts together with either metal salts or HBD. In contrast, types IV and V contain metal‐containing materials like hydrated metal chlorides and transition metals.^[^ ^19^ ^]^ Besides the principal types, there are developed subcategories focusing on pharmaceuticals. These are natural DES (NADES), which consists of endogenous metabolites like sugars, amino acids, and organic acids, along with THEDES, which are characterized by including APIs directly into the eutectic system.^[^ ^16^, ^20^ ^]^ Another significant one is hydrophobic DES (HDES), which is usually made from menthol and fatty acids or alcohols. These systems are particularly useful in improving the solubility and bioavailability of lipophilic drugs.^[^ ^21^ ^]^ **Figure** **1** offers a summary view integrating the various types of classifications, constituent parts of formulation science, and primary therapeutic uses of DES; it captures the essence of their structure and relevance in medicine and their engineering significance.

DES exhibit several distinctive physicochemical properties that set them apart from traditional solvents. Depending on their composition, they typically display high viscosity, low vapor pressure, and a broad polarity range. Their strong hydrogen‐bonding capability underpins their exceptional solvating power. Additionally, the biodegradability and low toxicity of many DES—especially those classified as NADES—enhance their appeal for biomedical applications.^[^ ^9^, ^10^ ^]^ Compared to conventional ionic liquids, DESs offer several advantages: they are generally easier to prepare, more cost‐effective, and more environmentally benign. Composed of readily available and pharmaceutically acceptable components, DESs often require no additional purification prior to use.^[^ ^22^, ^23^ ^]^

These advantageous properties have advanced DES's use in various pharmaceutical applications. Their remarkable solubilization capacity facilitates the dissolution of poorly water‐soluble APIs—such as ferulic acid, curcumin, and ibuprofen—often exceeding the performance of conventional organic solvents.^[^ ^24^, ^25^ ^]^ DES have also exhibited permeation‐enhancing effects, bioadhesive properties, and the ability to stabilize enzymes—highly relevant to transdermal, mucosal, and parenteral drug delivery applications.^[^ ^24^, ^26^ ^]^ In the case of THEDES, incorporating the API into the eutectic matrix allows the solvent to exhibit therapeutic functionality, resulting in dual‐action systems that offer synergistic therapeutic benefits.^[^ ^16^ ^]^

Nonetheless, several formulation challenges remain. The high viscosity of some DES formulations can impede handling and limit diffusion within specific delivery systems, while their hygroscopic nature may adversely affect the stability of the final product.^[^ ^7^, ^10^ ^]^ Potential incompatibilities between DES and specific polymers or APIs must be thoroughly evaluated. Nevertheless, the inherent modularity, sustainability, and capacity of DES to modulate matrix behavior continue to position them as a promising area of investigation in drug delivery research—particularly within polymer‐based systems, where they can concurrently influence both drug release kinetics and the structural properties of the delivery matrix.

Most pharmaceutical applications of DES fall predominantly within Type III, which involve combinations of quaternary ammonium salts such as choline chloride with organic HBD like urea, glycerol, or lactic acid. These types are especially favored due to their biocompatibility, low toxicity, and ability to solubilize hydrophilic and lipophilic compounds. Meanwhile, Type V DES, often referred to as THEDES, incorporates APIs directly as one‐of‐the‐components, enabling them to serve dual roles as both solvents and bioactive agents. By focusing on these pharmaceutically relevant types—Type III and V—the field continues to advance green, multifunctional, and customizable solvent systems for drug delivery applications. The schematic illustration provided in Figure 1 integrates this application‐focused classification with representative drugs and pharmaceutical purposes.

## 3 Polymeric Drug Carriers: Systems, Structure, and Release Mechanisms

Polymeric drug carriers have emerged as essential components in contemporary pharmaceutical formulations, owing to their structural versatility, mechanical adaptability, and ability to sustain and control drug release over extended periods. Formed from natural or synthetic products, these polymers form matrices that can encapsulate therapeutic agents and release them in a controlled manner either due to environmental stimuli or through natural degradation pathways. Polymers do not only serve as delivery vehicles; they also act as platforms that modulate the bioavailability and pharmacokinetics of the therapeutic agent and its targeting specificity with a broad range of therapeutic agents.^[^ ^27^, ^28^ ^]^

**Figure 2:**
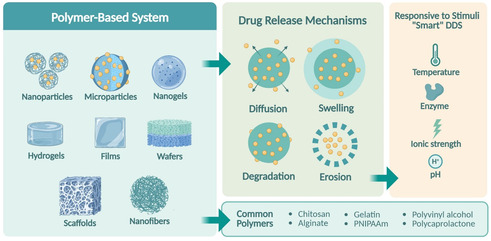
Overview of polymer‐based DDS, common polymers used, release mechanisms, and stimuli‐responsive features of “smart“ DDS. Created in BioRender. Nining, N. (2025) https://BioRender.com/unhta2f.

DDS utilizing polymers as the primary material can be subdivided into some groups, namely: particulate systems like nanoparticles and microparticles; gel‐based systems including hydrogels and nanogels; films and wafers, scaffolds as well as implantable devices.^[^ ^29^ ^]^ These polymer‐based DDS platforms, along with their most common polymers and potential responsiveness to stimuli, are summarized in **Figure** **2** to provide a more integrated view of their structural‐functional classification. Every method of drug delivery comes with its own set of benefits depending on the physical and chemical properties of the drug and its application in therapy. A good example would be nanoparticles, which can traverse biological boundaries and enable the precise delivery of therapeutics.^[^ ^30^ ^]^ Simultaneously, hydrogels possess high water content and excellent biocompatibility, rendering them particularly suitable for tissue‐compatible applications, including wound healing and ocular therapies.^[^ ^31^ ^]^ Polymeric films and membranes have demonstrated significant potential in transdermal and mucosal drug delivery, offering controlled and localized release of therapeutic agents at the application site.^[^ ^32^ ^]^

Drug release from polymeric systems is influenced by multiple factors, including the chemical structure of the polymer, its degree of crosslinking, the balance between hydrophilicity and hydrophobicity, and interactions with the surrounding biological environment. In general, drug release proceeds through four principal mechanisms, as illustrated in Figure 2: 1) Diffusion‐controlled release, where the drug migrates through the polymer matrix; 2) swelling‐controlled release, particularly in hydrophilic polymers that swell upon hydration; 3) degradation‐controlled release, involving enzymatic or hydrolytic breakdown of the polymer chains; and 4) erosion‐controlled release may occur at the surface or throughout the bulk of the material.^[^ ^33^ ^]^

The structural design of these carriers plays a crucial role in modulating drug release kinetics and determining their mechanical strength, biocompatibility, and compatibility with other formulation components.^[^ ^34^ ^]^ Parameters such as porosity, crosslinking density, polymer crystallinity, and drug‐polymer affinity influence how tightly the drug is retained within the matrix and how it is released in vivo.^[^ ^35^ ^]^ Formulating these structural features allows formulators to optimize the therapeutic profile and delivery route.

Polymers can be strategically engineered to respond to external stimuli—such as pH, temperature, ionic strength, or enzymatic activity—resulting in stimuli‐responsive or 'smart’ systems that enable on‐demand drug release.^[^ ^36^ ^]^ For instance, chitosan and alginate display pH‐responsive swelling behavior, which makes them well‐suited for applications in oral or colon‐targeted drug delivery systems.^[^ ^37^ ^]^ Thermoresponsive polymers, such as poly(N‐isopropyl acrylamide) (PNIPAAm), offer temperature‐sensitive drug release, enabling controlled delivery in response to thermal stimuli.^[^ ^38^ ^]^

Notably, incorporating DES into polymer systems—further explored in the following section—can lead to significant modifications in structural and functional properties. Acting as polymerizable solvents and functional media, DES can enhance the physicochemical characteristics of the polymer matrix while enabling precise control over drug loading and release in DDS.^[^ ^39^ ^]^

## 4 Functional Integration of DES into Polymeric Matrices

The incorporation of DES into polymer‐based DDS has opened new possibilities for fine‐tuning material properties and drug release profiles without necessitating chemical modification of the base polymer. Depending on their chemical nature and compatibility with the polymer matrix, DES can be introduced through various approaches, including direct polymerization, physical modification, and blending techniques.^[^ ^40^, ^41^ ^]^

The most important effect of including DES in the polymer matrix is their function as plasticizers, which reduces the glass transition temperature (Tg), increases molecular motion, and enhances the flexibility of the polymer matrix.^[^ ^39^ ^]^ For example, choline chloride‐based DESs have been reported to relax the rigidity of chitosan films, which increases their flexibility and possibly modifies their water vapor permeability.^[^ ^42^, ^43^ ^]^ The beneficial plasticizing effect of DES may be advantageous in drug delivery applications because it improves the pliability of the film and allows finer control over the release rate of the active drugs.

In addition to changing the physical form, DES also acts as a microstructural modulator by changing porosity, swelling characteristics, and changes in the molecular bonding of the material. For instance, introducing hydrophilic DES enhances the water affinity and swelling capacity of some hydrogels and scaffolds, especially those composed of natural polymers such as gelatin and alginate. This augmentation leads to improved drug release performance, which is triggered by swelling.^[^ ^44^ ^]^ Conversely, DES has a pronounced solvation capability for lipophilic compounds, which may improve their permittivity while simultaneously modulating release profiles for drug delivery.^[^ ^26^ ^]^

Another important aspect of DES is its capability to modify polymer‐drug interrelations. Due to their adjustable hydrogen bonding and polarity, DES can modulate the affinity of drug molecules to bond with the polymer matrix, either disrupting interactions for release or reinforcing interactions for sustained delivery, depending on the formulation strategy.^[^ ^6^ ^]^ Such versatility makes DES particularly appealing for controlling drug release behavior by integrating sophisticated temporal and spatial resolution via defined molecular interactions with responsive stimuli polymeric matrices.^[^ ^24^ ^]^ As noted, DES has been demonstrated to enhance drug solubility and improve oral bioavailability, which subsequently alters the pharmacokinetic behavior of drugs in polymeric DDS.^[^ ^45^ ^]^

THEDES showcase multifunctionality, where some act as solubilizing agents and as bioactive coformulants. An illustrative case is choline chloride and mandelic acid‐based THEDES, which have been successfully incorporated into electrospun gelatin fibers, yielding fast‐dissolving delivery systems with embedded antimicrobial activity.^[^ ^46^ ^]^ This collaborative approach allows for a reduction in the quantity of excipients required without compromising the therapeutic effectiveness of the formulation.

From a formulation standpoint, ensuring physicochemical compatibility between DES and the polymer matrix is essential for successful system performance. Key factors such as polarity alignment, hydrogen‐bonding interactions, and viscosity must be carefully considered to avoid issues like phase separation, undesired crosslinking, or compromised mechanical integrity. Research indicates that moderate polymer concentrations—typically around 30 wt%—within HIDES gel systems often strike an effective balance between mechanical strength and functional performance.^[^ ^47^ ^]^

In summary, integrating DES into polymeric DDS offers a versatile approach to tailoring matrix properties, modulating drug–carrier interactions, and fine‐tuning release kinetics—all within the framework of green chemistry. This functional incorporation of DES represents a forward‐looking advancement at the intersection of materials science and pharmaceutical technology, potentially transforming conventional polymers into high‐performance, next‐generation delivery platforms.

## 5 Impact of DES on Drug Release Profiles: Evidence from Literature

DES has shown a notable influence on drug release behavior within delivery systems, as evidenced by a range of release patterns and mechanisms outlined in **Table** **1**. Most studies report sustained‐release profiles, with fifteen investigations confirming this characteristic across various polymeric platforms. Moreover, the observed release behaviors exhibit considerable diversity, encompassing pH‐responsive release and initial burst effects.^[^ ^48^, ^49^, ^50^, ^51^, ^52^ ^]^

**Table 1:** Selected literature on DES–polymer systems and their effects on drug release.

Delivery system	Type of DES	Drug	Key Release Effect	Ref.
Xanthan gum supramolecular eutectogel	NADES (ChCl: citric/malic/lactic acid)	Benznidazole	Sustained, supersaturated release; viscosity‐dependent	[54]
Alginate‐chitosan hydrogel beads	DES (ChCl: glycerol)	Curcumin	pH‐responsive, sustained; burst in simulated intestinal fluid/simulated colonic fluid	[48]
Polyvinyl alcohol eutectogel	THEDES (Imidazole + ChCl/tetra butyl ammonium bromide/tetra butyl phosphonium bromide)	5‐Fluorouracil	pH‐responsive, sustained	[49]
Polycaprolactone matrix	THEDES (Metronidazole: maleic acid)	Metronidazole	Initial burst, then sustained (zero‐order)	[52]
Gelatin eutectogel	DES (ChCl: glycerol)	Curcumin (nanocrystal)	Sustained	[55]
Metal‐organic framework‐based molecularly imprinted polymer	DES (ChCl: methacrylic acid)	Doxorubicin, Phycocyanin	pH‐responsive, sustained	[50]
Polyvinyl alcohol nanofibers	DES (ChCl: urea)	Tetracycline	Alignment promoter and controlled drug release	[84]
Chitosan eutectogel	DES ((2‐Hydroxypropyl)‐β‐cyclodextrin: levulinic acid)	Resveratrol	Sustained	[57]
Poly(acrylic/ methacrylic acid)	THEDES (Lidocaine hydrochloride: acrylic/methacrylic acid)	Lidocaine hydrochloride	Sustained, pH/ionic strength‐controlled	[85]
Starch: polycaprolactone	THEDES (Menthol: ibuprofen)	Ibuprofen	Sustained, diffusion‐controlled	[59]
Agarose gel	DES (ChCl: malic acid)	Indomethacin, dexamethasone	Electro‐responsive, sustained	[58]
Starch: polycaprolactone	DES (ChCl: ascorbic acid)	Dexamethasone	Sustained	[56]
Gelatin bioplastic	DES (ChCl: glycerol) THEDES (Imipramine hydrochloride: glycerol)	Imipramine hydrochloride	Fast, transdermal	[51]
Carbomer 940 eutectogel	DES (ChCl: oxalic/malic/gallic acid)	Isoliquiritigenin	First‐order, sustained	[86]
Polycaprolactone nanofibres	DES (ChCl: acetic acid/glycerol)	Ibuprofen	Sustained, Fickian	[71]
Electrospun gelatin fibers	THEDES (ChCl: mandelic acid)	Mandelic acid	Fast‐dissolving	[87]
Polyvinyl alcohol/ lignocellulose nanofibril hydrogel	DES (ChCl: lactic acid)	Tetracycline hydrochloride	pH‐responsive, sustained, Fickian	[51]

Drug release durations across DES‐based systems vary widely, spanning from immediate release to prolonged delivery over several weeks. While specific formulations enable rapid release within minutes, others are designed to sustain drug release for up to 24 h or longer, depending on the therapeutic goals and system architecture.^[^ ^48^, ^53^, ^54^, ^55^, ^56^ ^]^ This flexibility in controlling release duration renders DES‐based systems highly adaptable to various therapeutic requirements.

The impact of DES on drug delivery is especially remarkable, particularly in terms of solubility enhancement. Several studies have documented substantial improvements, with some reporting up to a 36‐fold increase—and in exceptional cases, solubility enhancements surpassing 10 000‐fold—demonstrating the powerful solubilizing capacity of DES‐based systems.^[^ ^54^, ^57^ ^]^ Improvements in bioavailability have also been observed, with some studies reporting enhancements of up to 2.6‐fold, highlighting the potential of DES‐based systems to improve drug absorption and therapeutic efficacy 53 significantly.^[^ ^54^ ^]^ Numerous systems have achieved high cumulative drug release rates, typically between 78% and 97%, underscoring the effectiveness of DES‐based delivery platforms in facilitating efficient drug release.^[^ ^58^ ^]^

Within DDS, DES operate through multiple mechanisms, acting as solubilizing agents, plasticizers, crosslinkers, and structural network modifiers.^[^ ^48^, ^49^, ^57^, ^58^ ^]^ These underlying mechanisms are key in shaping distinct system characteristics, such as developing porous architectures, improved flexibility and adhesive properties, and self‐healing functionality.^[^ ^48^, ^49^, ^51^, ^55^, ^59^ ^]^ Certain systems also demonstrate enhanced electrical conductivity, introducing an additional functional attribute that may broaden their potential applications.^[^ ^58^ ^]^

These multifaceted properties position DES–polymer matrices as highly adaptable platforms for advanced pharmaceutical applications. For example, hydrophilic DES integrated into natural polymers like chitosan or alginate have been explored for transdermal patches and mucoadhesive films, offering improved solubility and permeation enhancement.^[^ ^60^, ^61^ ^]^ Thermoresponsive DES–polymer formulations can be engineered as injectable depots or in situ forming gels, enabling sustained release and localized delivery.^[^ ^49^, ^62^ ^]^ In wound healing, NADES with inherent antimicrobial activity can be embedded into polymeric scaffolds to promote tissue regeneration and infection control.^[^ ^14^, ^63^, ^64^ ^]^ Moreover, the plasticizing effect of DES facilitates low‐temperature 3D printing of customized drug delivery devices and flexible implants.^[^ ^65^, ^66^ ^]^ Their ionic nature also enhances the performance of electroconductive systems for stimuli‐responsive or smart DDS, such as in neural interfaces or controlled release under electric fields.^[^ ^44^, ^67^ ^]^

Eutectogels are the most studied and were included in five different studies. At the same time, this body of research also covers a range of other drug delivery platforms, such as hydrogels, bioplastics, nanofibers, metal‐organic frameworks, and polymer matrices. Although these systems differ in structure and composition, their modification from a structural and physicochemical perspective affects both release behavior and therapeutic efficacy, posing some advantages for drug delivery. Regarding DDS, the DES versatility across these platforms has been most helpful in enhancing drug solubility and controlled release, exemplifying their role as pivotal elements toward novel technologies in DDS.

## 6 Biopharmaceutical and Material Considerations

**Figure 3:**
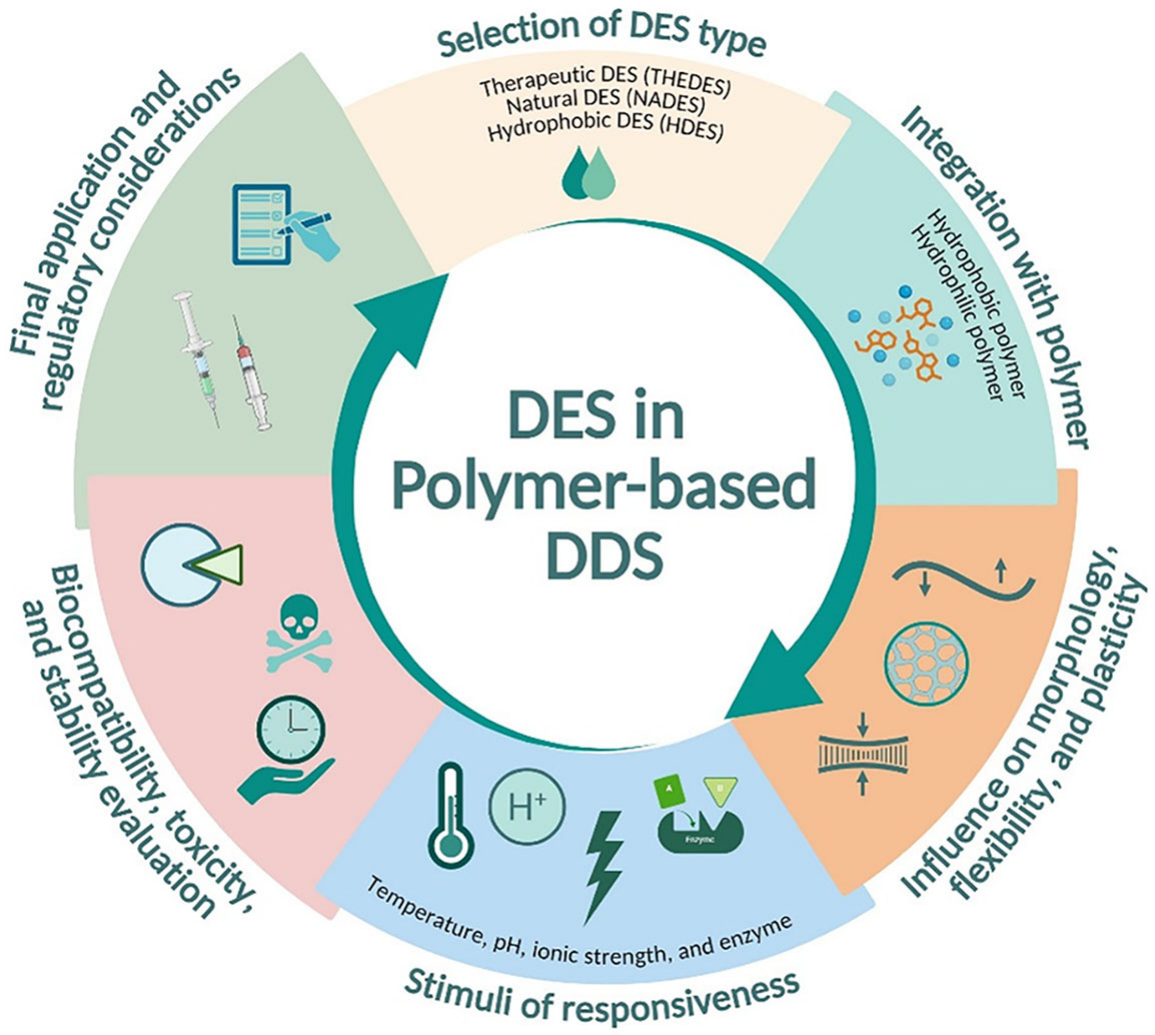
Key considerations for designing DES–polymer DDS include highlighting biocompatibility, morphology modulation, manufacturing stability, and regulatory factors, including NADES and THEDES components. Created in BioRender. Nining, N. (2025) https://BioRender.com/kdqcsih.

Incorporating DES into polymer‐based DDS requires consideration of more than just physicochemical and kinetic parameters. It also warrants biopharmaceutical evaluation for biocompatibility, cytotoxicity, formulation stability, and manufacturability. While enhancing solubility and modulating drug release are clear benefits of DES, their successful pharmaceutical application relies on understanding their long‐term interplay with biological systems and the polymer matrix (**Figure** **3**).

A focal point is the biocompatibility in the case of DES, not to mention when the polymer matrix is altered destructively for those particular solvents. Several formulations of DES, particularly NADES, which are built from choline chloride‐sugars and some other organic acids, are regarded as having low toxicity and good biocompatibility, mitigating in vitro and in vivo studies.^[^ ^68^, ^69^ ^]^ As an illustration, dose‐dependent effects have been documented. For instance, DES characterized by heightened viscosity or potent osmotic activity stands to risk damaging the cellular membrane or disturbing local hydration gradients if improperly concentrated during formulation.^[^ ^70^ ^]^ Therefore, it is necessary to establish suitable concentration thresholds during the formulation development stage to avoid the possibility of local irritations or toxic responses, especially in the case of parenteral and transdermal systems.

Beyond chemical composition, the morphology of polymer‐based DDS plays a critical role in determining drug release kinetics, mechanical behavior, and biological interaction. The incorporation of DES can significantly modulate morphological features such as porosity, surface texture, swelling behavior, and fiber alignment, depending on the formulation and processing method used. For instance, hydrophilic DES can modulate hydrogel morphology by inducing smaller, uniform pores or enlarged porous networks, depending on formulation conditions. These changes enhance flexibility, water uptake, and drug diffusion, improving permeability and tissue hydration.^[^ ^44^, ^60^ ^]^ In electrospun systems, the addition of DES such as choline chloride/acetic acid or choline chloride/glycerol can promote changes in nanofiber morphology and hydrophilicity, which improves fiber structure and maintains sustained drug release behavior without significantly affecting drug adsorption capacity.^[^ ^71^ ^]^ DES‐induced plasticization and surface modification can result in rougher or smoother morphologies across different polymers, enhancing flexibility, adhesion, and mechanical integrity for applications such as transdermal patches, mucoadhesive films, and tissue engineering scaffolds.^[^ ^72^, ^73^, ^74^ ^]^ These morphological changes are not merely incidental but often critical to the performance and functionality of the delivery system, making DES a powerful tool for fine‐tuning structure and therapeutic effect.

Ensuring long‐term stability presents an important problem when employing DES–polymer matrices. As DESs are hygroscopic, they absorb moisture that might substantially alter their physicochemical properties, such as viscosity, polarity, solubility, and phase behavior over time.^[^ ^6^, ^75^ ^]^ Research suggests that the compositions utilizing DES may undergo a form of physical softening or tackiness when exposed to heat or mechanical stress, especially if the DES is hydrophilic.^[^ ^76^ ^]^ To maintain the integrity of the product, it may be necessary to employ adequate packaging techniques and matrix reinforcement strategies like crosslinking or multilayer construction. In addition, stability, compatibility with sterilization procedures, and biological safety of the formulations containing DES need to be stringently evaluated. Processing conditions like autoclaving could subject DES to thermal degradation, compositional shifts, or even toxic interactions, necessitating rigorous evaluation during formulation development.^[^ ^77^, ^78^ ^]^ These factors introduce additional complexity, particularly for injectable or implantable applications requiring stringent aseptic assurance.

From a materials engineering perspective, DES can significantly influence polymer rheology, processability, and film‐forming behavior, facilitating innovative manufacturing approaches such as 3D printing, extrusion, and solvent‐free casting. The inherent plasticizing effect of DES is particularly advantageous for low‐temperature processing, as it reduces energy requirements and broadens the applicability of thermally sensitive APIs.^[^ ^13^ ^]^ However, excessive plasticization may lead to a reduction in tensile strength and an increase in elasticity, thereby limiting the suitability of such systems for load‐bearing or structurally demanding implant applications.^[^ ^42^ ^]^

Regulatory considerations become particularly pertinent when DES is combined with polymeric systems. Although many individual DES components are classified as generally recognized as safe (GRAS), the regulatory status of the eutectic mixture itself—especially in the case of THEDES containing pharmacologically active compounds—remains ambiguous and necessitates further evaluation across most regulatory jurisdictions.^[^ ^16^, ^45^ ^]^ Therefore, comprehensive toxicological and pharmacokinetic profiling is imperative for each DES–polymer‐drug formulation to ensure safety and therapeutic efficacy, particularly when systemic absorption is anticipated.

A critical distinction must be made between conventional DES and THEDES in the context of DDSs. While both share similar physicochemical properties—low volatility, high solubilizing capacity, and adjustable polarity—THEDES are uniquely formulated by combining eutectic components with APIs, imparting pharmacological activity to the solvent phase.^[^ ^11^, ^13^, ^16^, ^46^ ^]^ This dual‐functionality offers several advantages, such as simplified formulations, enhanced bioavailability, and synergistic therapeutic effects. For instance, THEDES, which are composed of ibuprofen or lidocaine with HBDs like menthol or lactic acid, can improve transdermal penetration while delivering therapeutic action.^[^ ^11^, ^13^, ^16^ ^]^ In contrast, conventional DES—typically composed of excipients such as choline chloride, urea, or sugars—function primarily as solubilizers, permeation enhancers, plasticizers, or stabilizers without intrinsic therapeutic effects.^[^ ^11^, ^67^ ^]^ The choice between DES and THEDES should thus be guided by the targeted delivery route, desired release profile, and regulatory considerations. THEDES offer exciting prospects for codelivery and dual‐action systems but also require more complex safety, toxicity, and pharmacokinetic evaluations, given that the API is a part of the solvent system.^[^ ^12^, ^13^, ^16^ ^]^

Despite these challenges, DES–polymer systems present notable advantages for site‐specific drug delivery, controlled release, and formulation simplification—particularly in scenarios where multiple excipients can be substituted with a single eutectic phase. Due to their multifunctional properties—including solubilization, plasticization, permeation enhancement, and modulation of drug release kinetics—DES are emerging as key enablers in developing next‐generation pharmaceutical platforms. To advance their clinical translation, ongoing research must prioritize the establishment of standardized protocols, comprehensive safety evaluations, and clear regulatory frameworks.

Understanding which DES types offer the best safety–performance balance is imperative to optimizing DES‐polymer DDS further. Based on current research, naturally derived NADES (such as those composed of choline chloride with sugars, amino acids, or organic acids) have shown superior biocompatibility and are frequently explored in pharmaceutical settings due to their low toxicity and GRAS‐status components.^[^ ^68^, ^69^, ^79^ ^]^ THEDES formulated with APIs show promise for dual‐functionality, acting both as drug solvents and active agents.^[^ ^11^, ^80^ ^]^ Hydrophilic DES are often better suited for hydrophilic polymers such as cellulose and polysaccharides, which belong to natural polymers. At the same time, lipophilic DES may be more compatible with hydrophobic matrices like polycaprolactone and nonpolar synthetic aliphatic polymers.^[^ ^39^, ^41^, ^81^ ^]^ Hence, rational selection of DES types based on polarity, component origin, and intended application route (e.g., transdermal, oral, and injectable) will enhance formulation performance and accelerate clinical translation.

## 7 Challenges, Limitations, and Opportunities

Although there is increasing interest in the broader use of DES in polymeric DDS, several unresolved limitations still constrain its use. Compromised formulation compatibility, stability of drug structure, regulatory ambiguity, and limited scope for upscaling production processes are all obstacles. Overcoming these challenges requires a multidisciplinary approach integrating applied and fundamental research.

One of the problems with DES‐polymer systems is the unpredictability of their interactions in multicomponent systems, perhaps in platforms that concern complex DDS. While issues with polymer integrity, drug compatibility, and release kinetics of DES are perplexing, they are of concern because of the easily adjustable nature of DES. The described discrepancy highlights the importance of reliable outcome formulation validation, which could be expected from systematic studies and model predictions. With a focus on one point, even if some combinations of DES and THEDES have shown good abilities to solubilize and enhance permeability, the solubility versatility paradox combined with strong covalent bonding poses significant risks to formulation stability besides influencing the rate of drug release and/or phase separation.^[^ ^24^, ^82^ ^]^

Furthermore, the lack of detailed structure‐activity relationship models limits forecasting changes in polymer morphology and drug release kinetic mechanisms regarding composition changes in DES, specifically alterations concerning the ratio of HBD to HBA.^[^ ^25^, ^83^ ^]^ Moisture uptake, oxidation, and phase change instability should be closely monitored, as they can negatively impact product reproducibility and shelf life. These issues highlight the necessity for comprehensive optimization strategies and in‐line analytical systems like rheometers and near‐infrared spectroscopy for real‐time assessment of DES–polymer compatibility.

From a regulatory viewpoint, most DES systems lie within a nebulous zone. While some constituents like choline chloride, lactic acid, and glycerol are considered GRAS, the eutectic blend is usually treated as a novel chemical entity, attracting greater regulatory concern. No clear guidance has been published by agencies like the FDA or EMA regarding DES and its use in pharmaceuticals, creating unanticipated timelines for approval. Such unclear guidelines weaken the confidence of industry stakeholders and create obstacles toward investment for developing and commercializing formulations based on DES.

However, these obstacles pose one‐of‐a‐kind chances for creativity. The configurational and adjustable characteristics of the DES provide an adaptable foundation for creating responsive and bespoke DDS targeting oncology, transdermal, mucosal, and implantable interfaces. THEDES, in which the solvent has biological activity, facilitate the development of dual‐action systems that function as carriers and cotherapeutic agents. These systems can streamline formulation, simplify therapeutic complexity, and/or reduce dosage while improving localized bioavailability.^[^ ^46^ ^]^

Additionally, integrating DES with emerging polymer technologies—such as stimuli‐responsive hydrogels, electrospun nanofibers, and 3D‐printed scaffolds—offers significant potential for developing next‐generation drug delivery platforms. Coupling these innovations with artificial intelligence and machine learning could enable exploring the vast combinatorial space of DES components, facilitating the creation of predictive models to optimize release kinetics, stability, and safety profiles. Such data‐driven approaches are expected to accelerate the rational design of formulations and their acceptance within regulatory frameworks.

In conclusion, despite existing limitations, DES–polymer systems hold significant transformative potential that warrants sustained interdisciplinary collaboration. Progress in computational modeling, high‐throughput formulation screening, and establishing standardized toxicological frameworks will be critical to fully realizing their value in advancing pharmaceutical innovation.

## 8 Future Perspectives

The progression of DES–polymer systems in drug delivery is highly contingent upon strategic integration across pharmaceutical sciences, materials engineering, and biotechnology. Future research must extend beyond in vitro investigations to facilitate clinical translation and prioritize in vivo studies that evaluate pharmacokinetics, biocompatibility, and therapeutic efficacy. Such evaluations are essential for validating DES‐integrated delivery systems’ safety and functional performance under physiologically relevant conditions.

Another critical avenue for advancement lies in integrating DES–polymer matrices with targeted delivery technologies, such as ligand‐mediated targeting, stimuli‐responsive systems, and programable implantable devices. By harnessing the tunable properties of DES, advanced drug delivery formulations have the potential to achieve not only controlled release but also precise spatial and temporal localization of therapeutics. In particular, THEDES present new opportunities for codelivery systems, wherein the solvent exerts pharmacological activity—thereby reducing formulation complexity and enhancing synergistic therapeutic effects.

From a regulatory perspective, the lack of standardized toxicological frameworks and classification systems for DES mixtures remains a significant barrier to pharmaceutical development. To facilitate their safe and effective integration, regulatory authorities such as the FDA and EMA must establish clear compositional guidelines and begin evaluating DES as distinct entities within the pharmaceutical landscape. To ensure the compatibility of DES with good manufacturing practice standards, comprehensive risk assessment protocols, long‐term stability evaluations, and robust quality control parameters must be developed alongside ongoing formulation research.

The progress made on DES–polymer platforms will, in the end, rely on strong collaborations across multiple academic fields. As the bioinspired design coupled with computational modeling and high‐throughput formulation screening works to characterize the vast compositional space of DESs, modeling their incorporation with diverse polymeric systems will also be crucial. Authorship was a collaborative effort, joining forces with technologies like 3D bioprinting, nanofabrication, and even machine learning, tailored biogenic responsive DES carriers can enable versatile adaptive, and sustainable personalized DDS.

## 9 Conclusion

The incorporation of DES into polymeric DDS heralds a novel translational method for cultivating multifunctional pharmaceutical formulations that are efficient and sustainable from an ecological perspective. DES exert profound influences on drug‐polymer interactions and drug release kinetics through numerous mechanisms such as enhancing solubility, hydrogen bond modification, and structural plasticization. Their increasing importance in developing advanced drug delivery systems is due to their adaptability, biodegradability, and dual function as a solvent and a therapeutic agent. This review presented fundamental considerations focusing on DES's composition and functional integration and their impact on the release profiles of drug‐loaded polymer matrices. Unresolved matters such as low reproducibility in formulating composition, regulatory ambiguity, lack of universally accepted standards for toxicological assessment, and an unyielding demand for thorough formulating reproducibility have also been outlined. Progress in this area will be achieved through strong interdisciplinary for interdisciplinary approaches, the use of accessible manufacturing systems, and the development of predictive frameworks for rational construction guidance. Collectively, polymer platforms incorporating DES offer significant potential to propel the development of next‐generation DDS that are personalized, programable, and responsive to biological cues.

## Conflict of Interest

The authors declare no conflict of interest.

## Supporting information

Supplementary Material
